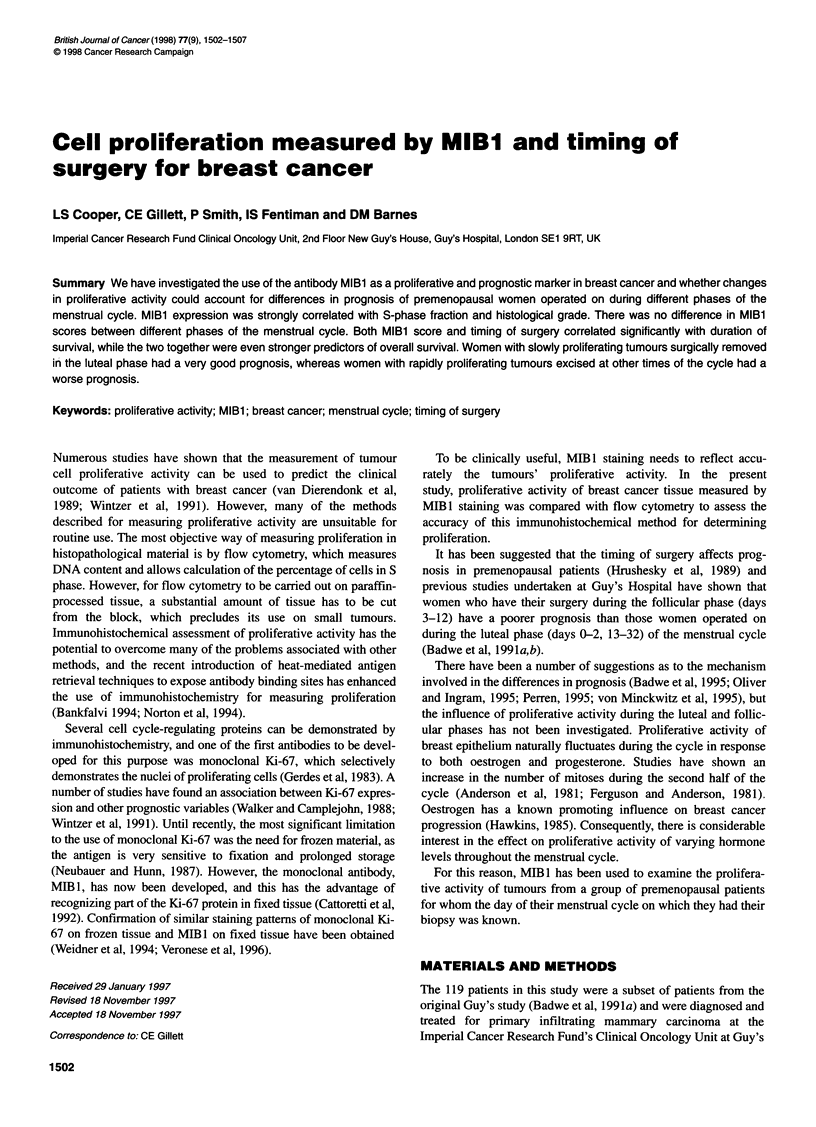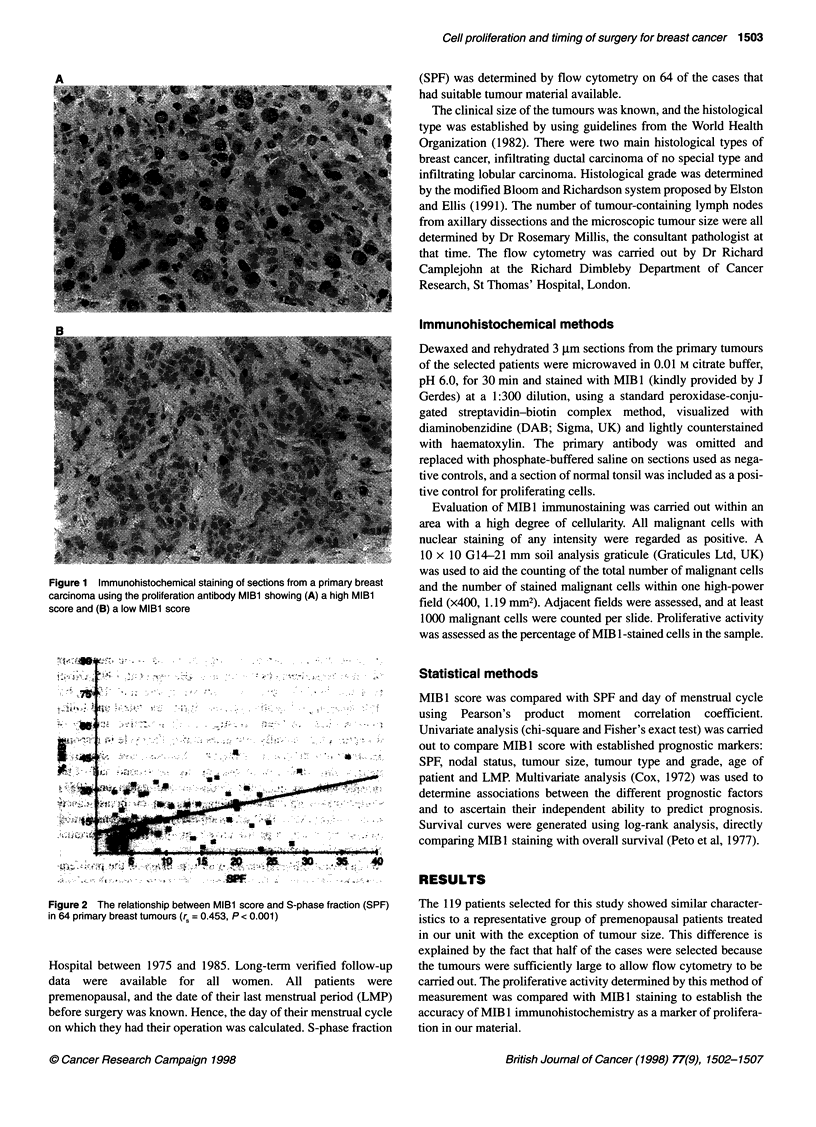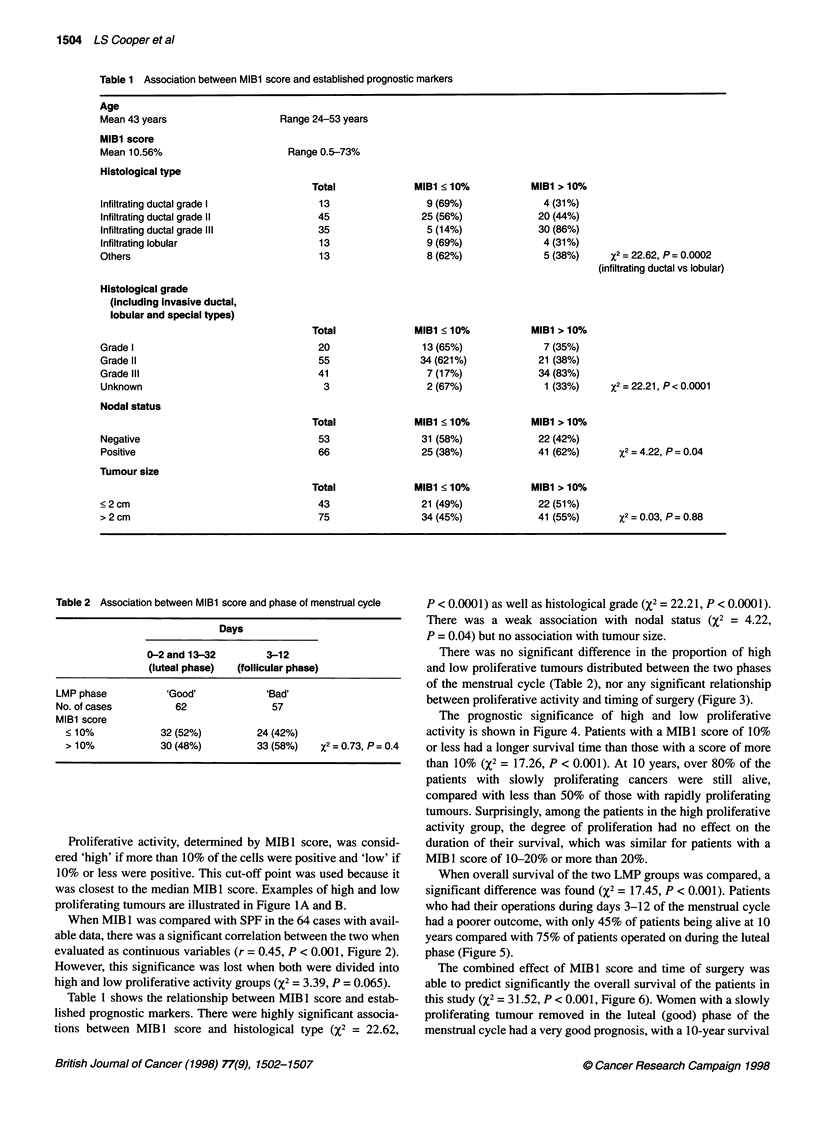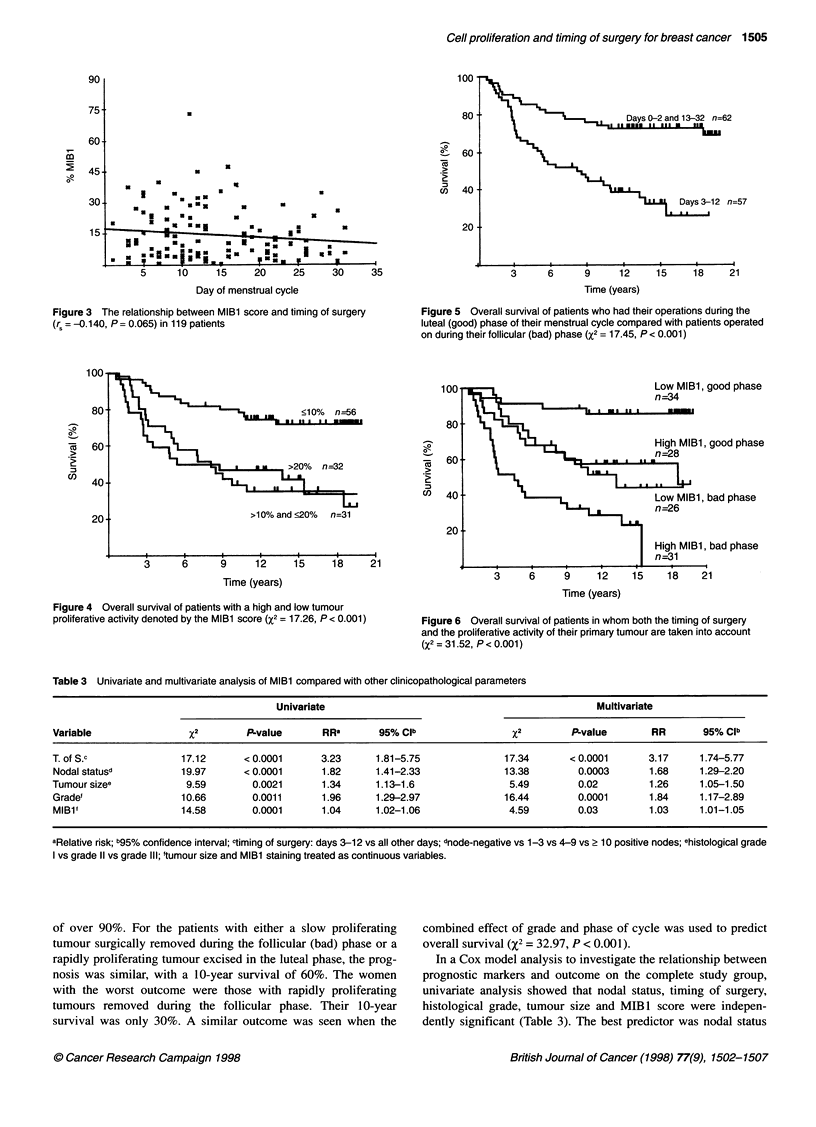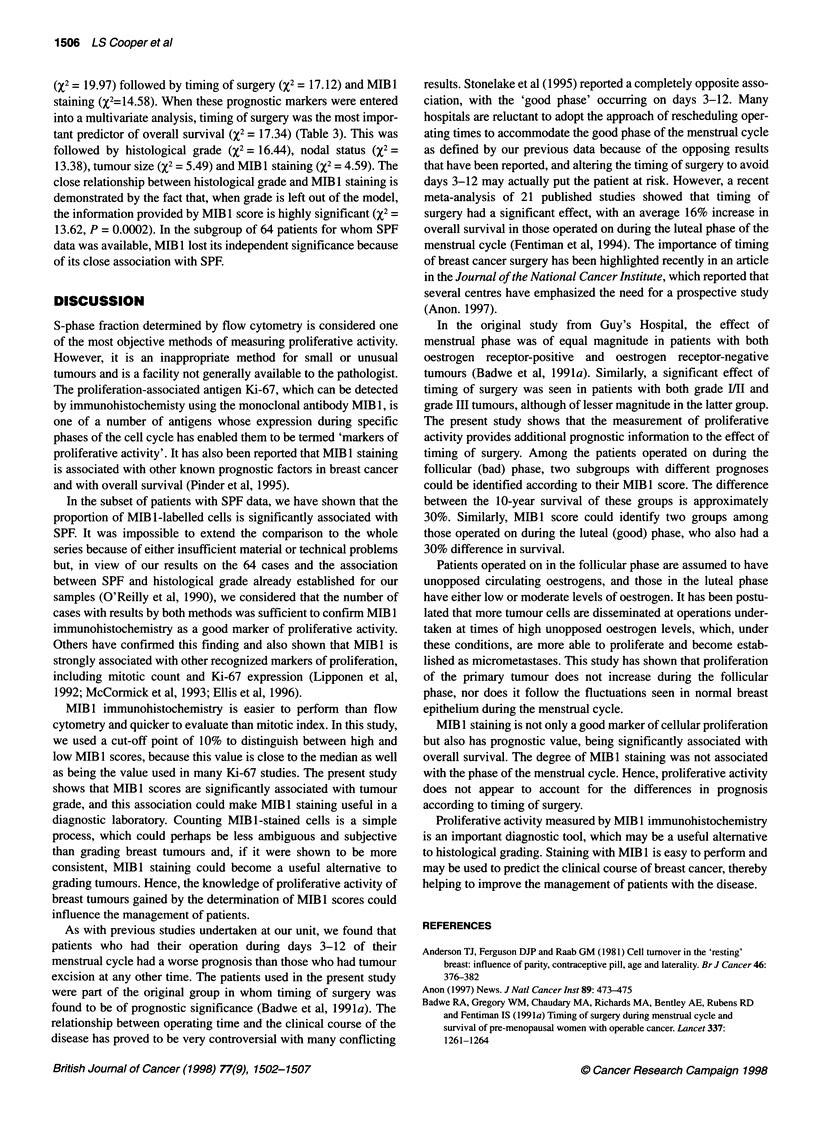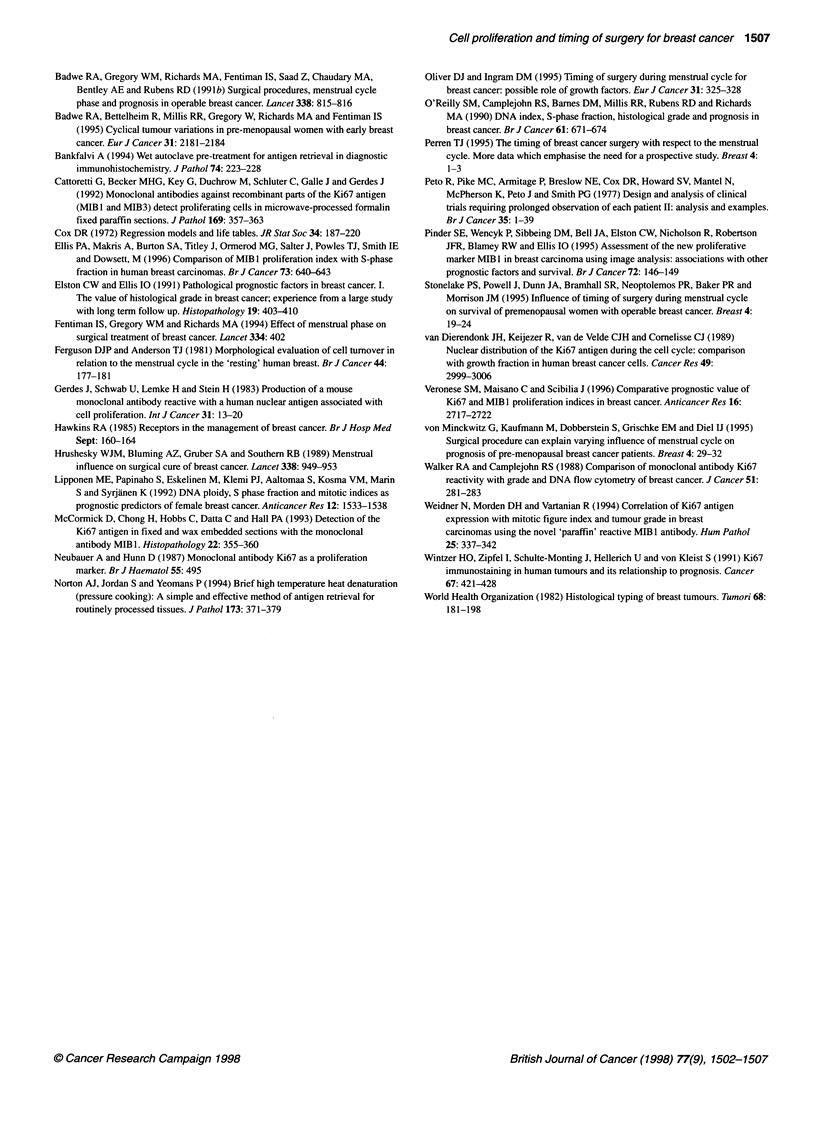# Cell proliferation measured by MIB1 and timing of surgery for breast cancer.

**DOI:** 10.1038/bjc.1998.247

**Published:** 1998-05

**Authors:** L. S. Cooper, C. E. Gillett, P. Smith, I. S. Fentiman, D. M. Barnes

**Affiliations:** Imperial Cancer Research Fund Clinical Oncology Unit, Guy's Hospital, London, UK.

## Abstract

**Images:**


British Journal of Cancer (1998) 77(9), 1502–1507
© 1998 Cancer Research Campaign

### Cell proliferation measured by MIB1 and timing of surgery for breast cancer

**LS Cooper, CE Gillett, P Smith, IS Fentiman and DM Barnes**

Imperial Cancer Research Fund Clinical Oncology Unit, 2nd Floor New Guy's House, Guy's Hospital, London SE1 9RT, UK

**Summary** We have investigated the use of the antibody MIB1 as a proliferative and prognostic marker in breast cancer and whether changes in proliferative activity could account for differences in prognosis of premenopausal women operated on during different phases of the menstrual cycle. MIB1 expression was strongly correlated with S-phase fraction and histological grade. There was no difference in MIB1 scores between different phases of the menstrual cycle. Both MIB1 score and timing of surgery correlated significantly with duration of survival, while the two together were even stronger predictors of overall survival. Women with slowly proliferating tumours surgically removed in the luteal phase had a very good prognosis, whereas women with rapidly proliferating tumours excised at other times of the cycle had a worse prognosis.

**Keywords:** proliferative activity; MIB1; breast cancer; menstrual cycle; timing of surgery

Numerous studies have shown that the measurement of tumour cell proliferative activity can be used to predict the clinical outcome of patients with breast cancer (van Dierendonk et al, 1989; Wintzer et al, 1991). However, many of the methods described for measuring proliferative activity are unsuitable for routine use. The most objective way of measuring proliferation in histopathological material is by flow cytometry, which measures DNA content and allows calculation of the percentage of cells in S phase. However, for flow cytometry to be carried out on paraffin-processed tissue, a substantial amount of tissue has to be cut from the block, which precludes its use on small tumours. Immunohistochemical assessment of proliferative activity has the potential to overcome many of the problems associated with other methods, and the recent introduction of heat-mediated antigen retrieval techniques to expose antibody binding sites has enhanced the use of immunohistochemistry for measuring proliferation (Bankfalvi 1994; Norton et al, 1994).

Several cell cycle-regulating proteins can be demonstrated by immunohistochemistry, and one of the first antibodies to be developed for this purpose was monoclonal Ki-67, which selectively demonstrates the nuclei of proliferating cells (Gerdes et al, 1983). A number of studies have found an association between Ki-67 expression and other prognostic variables (Walker and Camplejohn, 1988; Wintzer et al, 1991). Until recently, the most significant limitation to the use of monoclonal Ki-67 was the need for frozen material, as the antigen is very sensitive to fixation and prolonged storage (Neubauer and Hunn, 1987). However, the monoclonal antibody, MIB1, has now been developed, and this has the advantage of recognizing part of the Ki-67 protein in fixed tissue (Cattoretti et al, 1992). Confirmation of similar staining patterns of monoclonal Ki-67 on frozen tissue and MIB1 on fixed tissue have been obtained (Weidner et al, 1994; Veronese et al, 1996).

*Received 29 January 1997*
*Revised 18 November 1997*
*Accepted 18 November 1997*

*Correspondence to:* CE Gillett

To be clinically useful, MIB1 staining needs to reflect accurately the tumours' proliferative activity. In the present study, proliferative activity of breast cancer tissue measured by MIB1 staining was compared with flow cytometry to assess the accuracy of this immunohistochemical method for determining proliferation.

It has been suggested that the timing of surgery affects prognosis in premenopausal patients (Hrushesky et al, 1989) and previous studies undertaken at Guy's Hospital have shown that women who have their surgery during the follicular phase (days 3–12) have a poorer prognosis than those women operated on during the luteal phase (days 0–2, 13–32) of the menstrual cycle (Badwe et al, 1991*a,b*).

There have been a number of suggestions as to the mechanism involved in the differences in prognosis (Badwe et al, 1995; Oliver and Ingram, 1995; Perren, 1995; von Minckwitz et al, 1995), but the influence of proliferative activity during the luteal and follicular phases has not been investigated. Proliferative activity of breast epithelium naturally fluctuates during the cycle in response to both oestrogen and progesterone. Studies have shown an increase in the number of mitoses during the second half of the cycle (Anderson et al, 1981; Ferguson and Anderson, 1981). Oestrogen has a known promoting influence on breast cancer progression (Hawkins, 1985). Consequently, there is considerable interest in the effect on proliferative activity of varying hormone levels throughout the menstrual cycle.

For this reason, MIB1 has been used to examine the proliferative activity of tumours from a group of premenopausal patients for whom the day of their menstrual cycle on which they had their biopsy was known.

### MATERIALS AND METHODS

The 119 patients in this study were a subset of patients from the original Guy's study (Badwe et al, 1991*a*) and were diagnosed and treated for primary infiltrating mammary carcinoma at the Imperial Cancer Research Fund's Clinical Oncology Unit at Guy's

**Figure 1** Immunohistochemical staining of sections from a primary breast carcinoma using the proliferation antibody MIB1 showing (**A**) a high MIB1 score and (**B**) a low MIB1 score

**Figure 2** The relationship between MIB1 score and S-phase fraction (SPF) in 64 primary breast tumours ($r_s = 0.453$, $P < 0.001$)

Hospital between 1975 and 1985. Long-term verified follow-up data were available for all women. All patients were premenopausal, and the date of their last menstrual period (LMP) before surgery was known. Hence, the day of their menstrual cycle on which they had their operation was calculated. S-phase fraction (SPF) was determined by flow cytometry on 64 of the cases that had suitable tumour material available.

The clinical size of the tumours was known, and the histological type was established by using guidelines from the World Health Organization (1982). There were two main histological types of breast cancer, infiltrating ductal carcinoma of no special type and infiltrating lobular carcinoma. Histological grade was determined by the modified Bloom and Richardson system proposed by Elston and Ellis (1991). The number of tumour-containing lymph nodes from axillary dissections and the microscopic tumour size were all determined by Dr Rosemary Millis, the consultant pathologist at that time. The flow cytometry was carried out by Dr Richard Camplejohn at the Richard Dimbleby Department of Cancer Research, St Thomas' Hospital, London.

### Immunohistochemical methods

Dewaxed and rehydrated 3 μm sections from the primary tumours of the selected patients were microwaved in 0.01 M citrate buffer, pH 6.0, for 30 min and stained with MIB1 (kindly provided by J Gerdes) at a 1:300 dilution, using a standard peroxidase-conjugated streptavidin–biotin complex method, visualized with diaminobenzidine (DAB; Sigma, UK) and lightly counterstained with haematoxylin. The primary antibody was omitted and replaced with phosphate-buffered saline on sections used as negative controls, and a section of normal tonsil was included as a positive control for proliferating cells.

Evaluation of MIB1 immunostaining was carried out within an area with a high degree of cellularity. All malignant cells with nuclear staining of any intensity were regarded as positive. A 10 × 10 G14–21 mm soil analysis graticule (Graticules Ltd, UK) was used to aid the counting of the total number of malignant cells and the number of stained malignant cells within one high-power field (×400, 1.19 $mm^2$). Adjacent fields were assessed, and at least 1000 malignant cells were counted per slide. Proliferative activity was assessed as the percentage of MIB1-stained cells in the sample.

### Statistical methods

MIB1 score was compared with SPF and day of menstrual cycle using Pearson's product moment correlation coefficient. Univariate analysis (chi-square and Fisher's exact test) was carried out to compare MIB1 score with established prognostic markers: SPF, nodal status, tumour size, tumour type and grade, age of patient and LMP. Multivariate analysis (Cox, 1972) was used to determine associations between the different prognostic factors and to ascertain their independent ability to predict prognosis. Survival curves were generated using log-rank analysis, directly comparing MIB1 staining with overall survival (Peto et al, 1977).

### RESULTS

The 119 patients selected for this study showed similar characteristics to a representative group of premenopausal patients treated in our unit with the exception of tumour size. This difference is explained by the fact that half of the cases were selected because the tumours were sufficiently large to allow flow cytometry to be carried out. The proliferative activity determined by this method of measurement was compared with MIB1 staining to establish the accuracy of MIB1 immunohistochemistry as a marker of proliferation in our material.

© Cancer Research Campaign 1998

**Table 1** Association between MIB1 score and established prognostic markers

| | | | | |
|---|---|---|---|---|
| **Age** | | | | |
| Mean 43 years | Range 24–53 years | | | |
| **MIB1 score** | | | | |
| Mean 10.56% | Range 0.5–73% | | | |
| **Histological type** | | | | |
| | **Total** | **MIB1 ≤ 10%** | **MIB1 > 10%** | |
| Infiltrating ductal grade I | 13 | 9 (69%) | 4 (31%) | |
| Infiltrating ductal grade II | 45 | 25 (56%) | 20 (44%) | |
| Infiltrating ductal grade III | 35 | 5 (14%) | 30 (86%) | |
| Infiltrating lobular | 13 | 9 (69%) | 4 (31%) | |
| Others | 13 | 8 (62%) | 5 (38%) | $\chi^2 = 22.62$, $P = 0.0002$ (infiltrating ductal vs lobular) |
| **Histological grade (including invasive ductal, lobular and special types)** | | | | |
| | **Total** | **MIB1 ≤ 10%** | **MIB1 > 10%** | |
| Grade I | 20 | 13 (65%) | 7 (35%) | |
| Grade II | 55 | 34 (621%) | 21 (38%) | |
| Grade III | 41 | 7 (17%) | 34 (83%) | |
| Unknown | 3 | 2 (67%) | 1 (33%) | $\chi^2 = 22.21$, $P < 0.0001$ |
| **Nodal status** | | | | |
| | **Total** | **MIB1 ≤ 10%** | **MIB1 > 10%** | |
| Negative | 53 | 31 (58%) | 22 (42%) | |
| Positive | 66 | 25 (38%) | 41 (62%) | $\chi^2 = 4.22$, $P = 0.04$ |
| **Tumour size** | | | | |
| | **Total** | **MIB1 ≤ 10%** | **MIB1 > 10%** | |
| ≤ 2 cm | 43 | 21 (49%) | 22 (51%) | |
| > 2 cm | 75 | 34 (45%) | 41 (55%) | $\chi^2 = 0.03$, $P = 0.88$ |

**Table 2** Association between MIB1 score and phase of menstrual cycle

| | **Days** | | |
|---|---|---|---|
| | **0–2 and 13–32 (luteal phase)** | **3–12 (follicular phase)** | |
| LMP phase | 'Good' | 'Bad' | |
| No. of cases | 62 | 57 | |
| MIB1 score | | | |
| ≤ 10% | 32 (52%) | 24 (42%) | |
| > 10% | 30 (48%) | 33 (58%) | $\chi^2 = 0.73$, $P = 0.4$ |

Proliferative activity, determined by MIB1 score, was considered 'high' if more than 10% of the cells were positive and 'low' if 10% or less were positive. This cut-off point was used because it was closest to the median MIB1 score. Examples of high and low proliferating tumours are illustrated in Figure 1A and B.

When MIB1 was compared with SPF in the 64 cases with available data, there was a significant correlation between the two when evaluated as continuous variables ($r = 0.45$, $P < 0.001$, Figure 2). However, this significance was lost when both were divided into high and low proliferative activity groups ($\chi^2 = 3.39$, $P = 0.065$).

Table 1 shows the relationship between MIB1 score and established prognostic markers. There were highly significant associations between MIB1 score and histological type ($\chi^2 = 22.62$, $P < 0.0001$) as well as histological grade ($\chi^2 = 22.21$, $P < 0.0001$). There was a weak association with nodal status ($\chi^2 = 4.22$, $P = 0.04$) but no association with tumour size.

There was no significant difference in the proportion of high and low proliferative tumours distributed between the two phases of the menstrual cycle (Table 2), nor any significant relationship between proliferative activity and timing of surgery (Figure 3).

The prognostic significance of high and low proliferative activity is shown in Figure 4. Patients with a MIB1 score of 10% or less had a longer survival time than those with a score of more than 10% ($\chi^2 = 17.26$, $P < 0.001$). At 10 years, over 80% of the patients with slowly proliferating cancers were still alive, compared with less than 50% of those with rapidly proliferating tumours. Surprisingly, among the patients in the high proliferative activity group, the degree of proliferation had no effect on the duration of their survival, which was similar for patients with a MIB1 score of 10–20% or more than 20%.

When overall survival of the two LMP groups was compared, a significant difference was found ($\chi^2 = 17.45$, $P < 0.001$). Patients who had their operations during days 3–12 of the menstrual cycle had a poorer outcome, with only 45% of patients being alive at 10 years compared with 75% of patients operated on during the luteal phase (Figure 5).

The combined effect of MIB1 score and time of surgery was able to predict significantly the overall survival of the patients in this study ($\chi^2 = 31.52$, $P < 0.001$, Figure 6). Women with a slowly proliferating tumour removed in the luteal (good) phase of the menstrual cycle had a very good prognosis, with a 10-year survival

*© Cancer Research Campaign 1998*

**Figure 3** The relationship between MIB1 score and timing of surgery ($r_s$ = –0.140, *P* = 0.065) in 119 patients

**Figure 5** Overall survival of patients who had their operations during the luteal (good) phase of their menstrual cycle compared with patients operated on during their follicular (bad) phase ($\chi^2$ = 17.45, *P* < 0.001)

**Figure 4** Overall survival of patients with a high and low tumour proliferative activity denoted by the MIB1 score ($\chi^2$ = 17.26, *P* < 0.001)

**Figure 6** Overall survival of patients in whom both the timing of surgery and the proliferative activity of their primary tumour are taken into account ($\chi^2$ = 31.52, *P* < 0.001)

**Table 3** Univariate and multivariate analysis of MIB1 compared with other clinicopathological parameters

| | Univariate | | | | Multivariate | | | |
|---|---|---|---|---|---|---|---|---|
| **Variable** | $\chi^2$ | *P*-value | RR[a] | 95% CI[b] | $\chi^2$ | *P*-value | RR | 95% CI[b] |
| T. of S.[c] | 17.12 | < 0.0001 | 3.23 | 1.81–5.75 | 17.34 | < 0.0001 | 3.17 | 1.74–5.77 |
| Nodal status[d] | 19.97 | < 0.0001 | 1.82 | 1.41–2.33 | 13.38 | 0.0003 | 1.68 | 1.29–2.20 |
| Tumour size[e] | 9.59 | 0.0021 | 1.34 | 1.13–1.6 | 5.49 | 0.02 | 1.26 | 1.05–1.50 |
| Grade[f] | 10.66 | 0.0011 | 1.96 | 1.29–2.97 | 16.44 | 0.0001 | 1.84 | 1.17–2.89 |
| MIB1[f] | 14.58 | 0.0001 | 1.04 | 1.02–1.06 | 4.59 | 0.03 | 1.03 | 1.01–1.05 |

[a]Relative risk; [b]95% confidence interval; [c]timing of surgery: days 3–12 vs all other days; [d]node-negative vs 1–3 vs 4–9 vs ≥ 10 positive nodes; [e]histological grade I vs grade II vs grade III; [f]tumour size and MIB1 staining treated as continuous variables.

of over 90%. For the patients with either a slow proliferating tumour surgically removed during the follicular (bad) phase or a rapidly proliferating tumour excised in the luteal phase, the prognosis was similar, with a 10-year survival of 60%. The women with the worst outcome were those with rapidly proliferating tumours removed during the follicular phase. Their 10-year survival was only 30%. A similar outcome was seen when the combined effect of grade and phase of cycle was used to predict overall survival ($\chi^2$ = 32.97, *P* < 0.001).

In a Cox model analysis to investigate the relationship between prognostic markers and outcome on the complete study group, univariate analysis showed that nodal status, timing of surgery, histological grade, tumour size and MIB1 score were independently significant (Table 3). The best predictor was nodal status

($\chi^2$ = 19.97) followed by timing of surgery ($\chi^2$ = 17.12) and MIB1 staining ($\chi^2$=14.58). When these prognostic markers were entered into a multivariate analysis, timing of surgery was the most important predictor of overall survival ($\chi^2$ = 17.34) (Table 3). This was followed by histological grade ($\chi^2$ = 16.44), nodal status ($\chi^2$ = 13.38), tumour size ($\chi^2$ = 5.49) and MIB1 staining ($\chi^2$ = 4.59). The close relationship between histological grade and MIB1 staining is demonstrated by the fact that, when grade is left out of the model, the information provided by MIB1 score is highly significant ($\chi^2$ = 13.62, $P$ = 0.0002). In the subgroup of 64 patients for whom SPF data was available, MIB1 lost its independent significance because of its close association with SPF.

### DISCUSSION

S-phase fraction determined by flow cytometry is considered one of the most objective methods of measuring proliferative activity. However, it is an inappropriate method for small or unusual tumours and is a facility not generally available to the pathologist. The proliferation-associated antigen Ki-67, which can be detected by immunohistochemisty using the monoclonal antibody MIB1, is one of a number of antigens whose expression during specific phases of the cell cycle has enabled them to be termed 'markers of proliferative activity'. It has also been reported that MIB1 staining is associated with other known prognostic factors in breast cancer and with overall survival (Pinder et al, 1995).

In the subset of patients with SPF data, we have shown that the proportion of MIB1-labelled cells is significantly associated with SPF. It was impossible to extend the comparison to the whole series because of either insufficient material or technical problems but, in view of our results on the 64 cases and the association between SPF and histological grade already established for our samples (O'Reilly et al, 1990), we considered that the number of cases with results by both methods was sufficient to confirm MIB1 immunohistochemistry as a good marker of proliferative activity. Others have confirmed this finding and also shown that MIB1 is strongly associated with other recognized markers of proliferation, including mitotic count and Ki-67 expression (Lipponen et al, 1992; McCormick et al, 1993; Ellis et al, 1996).

MIB1 immunohistochemistry is easier to perform than flow cytometry and quicker to evaluate than mitotic index. In this study, we used a cut-off point of 10% to distinguish between high and low MIB1 scores, because this value is close to the median as well as being the value used in many Ki-67 studies. The present study shows that MIB1 scores are significantly associated with tumour grade, and this association could make MIB1 staining useful in a diagnostic laboratory. Counting MIB1-stained cells is a simple process, which could perhaps be less ambiguous and subjective than grading breast tumours and, if it were shown to be more consistent, MIB1 staining could become a useful alternative to grading tumours. Hence, the knowledge of proliferative activity of breast tumours gained by the determination of MIB1 scores could influence the management of patients.

As with previous studies undertaken at our unit, we found that patients who had their operation during days 3–12 of their menstrual cycle had a worse prognosis than those who had tumour excision at any other time. The patients used in the present study were part of the original group in whom timing of surgery was found to be of prognostic significance (Badwe et al, 1991*a*). The relationship between operating time and the clinical course of the disease has proved to be very controversial with many conflicting results. Stonelake et al (1995) reported a completely opposite association, with the 'good phase' occurring on days 3–12. Many hospitals are reluctant to adopt the approach of rescheduling operating times to accommodate the good phase of the menstrual cycle as defined by our previous data because of the opposing results that have been reported, and altering the timing of surgery to avoid days 3–12 may actually put the patient at risk. However, a recent meta-analysis of 21 published studies showed that timing of surgery had a significant effect, with an average 16% increase in overall survival in those operated on during the luteal phase of the menstrual cycle (Fentiman et al, 1994). The importance of timing of breast cancer surgery has been highlighted recently in an article in the *Journal of the National Cancer Institute*, which reported that several centres have emphasized the need for a prospective study (Anon. 1997).

In the original study from Guy's Hospital, the effect of menstrual phase was of equal magnitude in patients with both oestrogen receptor-positive and oestrogen receptor-negative tumours (Badwe et al, 1991*a*). Similarly, a significant effect of timing of surgery was seen in patients with both grade I/II and grade III tumours, although of lesser magnitude in the latter group. The present study shows that the measurement of proliferative activity provides additional prognostic information to the effect of timing of surgery. Among the patients operated on during the follicular (bad) phase, two subgroups with different prognoses could be identified according to their MIB1 score. The difference between the 10-year survival of these groups is approximately 30%. Similarly, MIB1 score could identify two groups among those operated on during the luteal (good) phase, who also had a 30% difference in survival.

Patients operated on in the follicular phase are assumed to have unopposed circulating oestrogens, and those in the luteal phase have either low or moderate levels of oestrogen. It has been postulated that more tumour cells are disseminated at operations undertaken at times of high unopposed oestrogen levels, which, under these conditions, are more able to proliferate and become established as micrometastases. This study has shown that proliferation of the primary tumour does not increase during the follicular phase, nor does it follow the fluctuations seen in normal breast epithelium during the menstrual cycle.

MIB1 staining is not only a good marker of cellular proliferation but also has prognostic value, being significantly associated with overall survival. The degree of MIB1 staining was not associated with the phase of the menstrual cycle. Hence, proliferative activity does not appear to account for the differences in prognosis according to timing of surgery.

Proliferative activity measured by MIB1 immunohistochemistry is an important diagnostic tool, which may be a useful alternative to histological grading. Staining with MIB1 is easy to perform and may be used to predict the clinical course of breast cancer, thereby helping to improve the management of patients with the disease.

### REFERENCES

Anderson TJ, Ferguson DJP and Raab GM (1981) Cell turnover in the 'resting' breast: influence of parity, contraceptive pill, age and laterality. *Br J Cancer* **46**: 376–382

Anon (1997) News. *J Natl Cancer Inst* **89**: 473–475

Badwe RA, Gregory WM, Chaudary MA, Richards MA, Bentley AE, Rubens RD and Fentiman IS (1991*a*) Timing of surgery during menstrual cycle and survival of pre-menopausal women with operable cancer. *Lancet* **337**: 1261–1264

© Cancer Research Campaign 1998

Badwe RA, Gregory WM, Richards MA, Fentiman IS, Saad Z, Chaudary MA, Bentley AE and Rubens RD (1991*b*) Surgical procedures, menstrual cycle phase and prognosis in operable breast cancer. *Lancet* **338**: 815–816

Badwe RA, Bettelheim R, Millis RR, Gregory W, Richards MA and Fentiman IS (1995) Cyclical tumour variations in pre-menopausal women with early breast cancer. *Eur J Cancer* **31**: 2181–2184

Bankfalvi A (1994) Wet autoclave pre-treatment for antigen retrieval in diagnostic immunohistochemistry. *J Pathol* **74**: 223–228

Cattoretti G, Becker MHG, Key G, Duchrow M, Schluter C, Galle J and Gerdes J (1992) Monoclonal antibodies against recombinant parts of the Ki67 antigen (MIB1 and MIB3) detect proliferating cells in microwave-processed formalin fixed paraffin sections. *J Pathol* **169**: 357–363

Cox DR (1972) Regression models and life tables. *JR Stat Soc* **34**: 187–220

Ellis PA, Makris A, Burton SA, Titley J, Ormerod MG, Salter J, Powles TJ, Smith IE and Dowsett, M (1996) Comparison of MIB1 proliferation index with S-phase fraction in human breast carcinomas. *Br J Cancer* **73**: 640–643

Elston CW and Ellis IO (1991) Pathological prognostic factors in breast cancer. I. The value of histological grade in breast cancer; experience from a large study with long term follow up. *Histopathology* **19**: 403–410

Fentiman IS, Gregory WM and Richards MA (1994) Effect of menstrual phase on surgical treatment of breast cancer. *Lancet* **334**: 402

Ferguson DJP and Anderson TJ (1981) Morphological evaluation of cell turnover in relation to the menstrual cycle in the 'resting' human breast. *Br J Cancer* **44**: 177–181

Gerdes J, Schwab U, Lemke H and Stein H (1983) Production of a mouse monoclonal antibody reactive with a human nuclear antigen associated with cell proliferation. *Int J Cancer* **31**: 13–20

Hawkins RA (1985) Receptors in the management of breast cancer. *Br J Hosp Med* **Sept**: 160–164

Hrushesky WJM, Bluming AZ, Gruber SA and Southern RB (1989) Menstrual influence on surgical cure of breast cancer. *Lancet* **338**: 949–953

Lipponen ME, Papinaho S, Eskelinen M, Klemi PJ, Aaltomaa S, Kosma VM, Marin S and Syrjänen K (1992) DNA ploidy, S phase fraction and mitotic indices as prognostic predictors of female breast cancer. *Anticancer Res* **12**: 1533–1538

McCormick D, Chong H, Hobbs C, Datta C and Hall PA (1993) Detection of the Ki67 antigen in fixed and wax embedded sections with the monoclonal antibody MIB1. *Histopathology* **22**: 355–360

Neubauer A and Hunn D (1987) Monoclonal antibody Ki67 as a proliferation marker. *Br J Haematol* **55**: 495

Norton AJ, Jordan S and Yeomans P (1994) Brief high temperature heat denaturation (pressure cooking): A simple and effective method of antigen retrieval for routinely processed tissues. *J Pathol* **173**: 371–379

Oliver DJ and Ingram DM (1995) Timing of surgery during menstrual cycle for breast cancer: possible role of growth factors. *Eur J Cancer* **31**: 325–328

O'Reilly SM, Camplejohn RS, Barnes DM, Millis RR, Rubens RD and Richards MA (1990) DNA index, S-phase fraction, histological grade and prognosis in breast cancer. *Br J Cancer* **61**: 671–674

Perren TJ (1995) The timing of breast cancer surgery with respect to the menstrual cycle. More data which emphasise the need for a prospective study. *Breast* **4**: 1–3

Peto R, Pike MC, Armitage P, Breslow NE, Cox DR, Howard SV, Mantel N, McPherson K, Peto J and Smith PG (1977) Design and analysis of clinical trials requiring prolonged observation of each patient II: analysis and examples. *Br J Cancer* **35**: 1–39

Pinder SE, Wencyk P, Sibbeing DM, Bell JA, Elston CW, Nicholson R, Robertson JFR, Blamey RW and Ellis IO (1995) Assessment of the new proliferative marker MIB1 in breast carcinoma using image analysis: associations with other prognostic factors and survival. *Br J Cancer* **72**: 146–149

Stonelake PS, Powell J, Dunn JA, Bramhall SR, Neoptolemos PR, Baker PR and Morrison JM (1995) Influence of timing of surgery during menstrual cycle on survival of premenopausal women with operable breast cancer. *Breast* **4**: 19–24

van Dierendonk JH, Keijezer R, van de Velde CJH and Cornelisse CJ (1989) Nuclear distribution of the Ki67 antigen during the cell cycle: comparison with growth fraction in human breast cancer cells. *Cancer Res* **49**: 2999–3006

Veronese SM, Maisano C and Scibilia J (1996) Comparative prognostic value of Ki67 and MIB1 proliferation indices in breast cancer. *Anticancer Res* **16**: 2717–2722

von Minckwitz G, Kaufmann M, Dobberstein S, Grischke EM and Diel IJ (1995) Surgical procedure can explain varying influence of menstrual cycle on prognosis of pre-menopausal breast cancer patients. *Breast* **4**: 29–32

Walker RA and Camplejohn RS (1988) Comparison of monoclonal antibody Ki67 reactivity with grade and DNA flow cytometry of breast cancer. *J Cancer* **51**: 281–283

Weidner N, Morden DH and Vartanian R (1994) Correlation of Ki67 antigen expression with mitotic figure index and tumour grade in breast carcinomas using the novel 'paraffin' reactive MIB1 antibody. *Hum Pathol* **25**: 337–342

Wintzer HO, Zipfel I, Schulte-Monting J, Hellerich U and von Kleist S (1991) Ki67 immunostaining in human tumours and its relationship to prognosis. *Cancer* **67**: 421–428

World Health Organization (1982) Histological typing of breast tumours. *Tumori* **68**: 181–198

© Cancer Research Campaign 1998